# Evaluation on Hope and Psychological Symptoms in Infertile Couples Undergoing Assisted Reproduction Treatment

**DOI:** 10.22074/ijfs.2017.4838

**Published:** 2017-02-16

**Authors:** Reza Omani Samani, Samira Vesali, Behnaz Navid, Bahareh Vakiliniya, Maryam Mohammadi

**Affiliations:** Department of Epidemiology and Reproductive Health, Reproductive Epidemiology Research Center, Royan Institute for Reproductive Biomedicine, ACECR, Tehran, Iran

**Keywords:** Hope, Depression, Anxiety, Stress, Infertility

## Abstract

**Background:**

This study evaluated hope, depression, anxiety, and stress among three groups of infertile couples.

**Materials and Methods:**

This cross-sectional study consisted of three groups of infertile couples-candidates for oocyte donation (n=60), embryo donation (n=60), and normal infertile (n=60). Participants included couples seen at Royan Institute, Tehran, Iran between 2013-2014 who were at least 18 years of age and could read and write in Persian. Participants provided demographic and general characteristics and completed the Persian version of the Adult Trait Hope Scale (hope, agency and pathway) and Depression, Anxiety, and Stress Scale (DASS). Data was analyzed by the paired t test, ANOVA, ANCOVA and Pearson correlation tests using SPSS statistical software.

**Results:**

Overall, 180 infertile couples participated in the three groups. There was a significant higher mean score for hope in husbands compared to wives in the normal infertile group (P=0.046). Husbands in the normal infertile group also had a significantly higher mean score for pathway (P=0.032). The frequency of anxiety significantly differed in female subjects (P=0.028). In the normal infertile group, the anxiety distribution significantly differed between wives and husbands (P=0.006). There was a significantly different stress frequency in male subjects (P=0.048). In the embryo donation group, stress significantly differed between wives and husbands (P=0.002). In the normal infertile group, stress also significantly differed between wives and husbands (P=0.05).

**Conclusion:**

The results have suggested that hope might be important in reducing psychological symptoms and psychological adjustment in those exposed to infertility problems who follow medical recommendations, which accelerates recovery. It is recommended to hold psychological counseling sessions (hope therapy) during reproduction cycles.

## Introduction

Infertility is an unpleasant, unexpected experience for individuals. According to a populationbased study in Iran, the overall prevalence of lifetime primary infertility among couples is 17.3% (1). Infertility is associated with tremendous negative psychological and mental burdens on both infertile men and women, in addition to somatic and sexual disorders (2). The typical, common psychological problems that result from infertility are anxiety, depression, low self-confidence, stress, distress, and lower marital and sexual satisfaction (3, 4). When infertility treatment takes a long time or in the event of treatment failure, infertile patients are more likely to encounter hopelessness. The loss of hope to have a child is important because hope is one of the main psychological needs (5). In other words, hope generates self-confidence and internal positive feelings toward solving an existing problem. Hope has been defined by Snyder et al. (6) as “a reciprocally derived sense of successful agency (goal-directed determination) and pathways (planning of ways to meet goals)”. What can be deduced is that agency is the perception that one can reach his/her goals, whereas pathways is known as the perception that one can find alternative routes to reach these goals should the need arise (7). It is expected that people with high levels of hope can think about pathways to reach their goals, deal with diseases better, and generate additional coping strategies (8) so that fear, anxiety, and fatigue occur less often in these individuals (9, 10). Hence, psychologists should find hope and belief issues in patients in order to facilitate the treatment process during a life crisis, because this is considered an important part of patient treatment (10).

To the best of our knowledge, no studies have examined the utility of Snyder’s theory of hope in patients who suffer from infertility. This study aimed to investigate the levels of hope as conceptualized by Snyder in infertile couples who undergo infertility treatment. Specifically, we sought to determine whether hope would be significantly positively or negatively related to major psychological symptoms commonly experienced by infertile patients (i.e., anxiety, stress, and depression).

## Materials and Methods

We conducted this cross-sectional study on infertile couples who referred to Royan Institute, a referral infertility clinic in Tehran, Iran between 2013 and 2014. The study sample consisted of three groups of infertile couples-candidates for oocyte donation (n=60), candidates for embryo donation (n=60), and normal infertile (n=60). The inclusion criteria were aged 18 years or older, a history of infertility, and ability to read and write in Persian.

The Ethical Committee of Royan Institute approved the study. Aims of the study and the confidentiality of the data were clearly explained for all participants. We reassured all eligible individuals that acceptance or refusal to participate in the research had no influence on their treatment procedures. Voluntary completion of the questionnaire was considered as written informed consent. Participants completed three questionnaires. First, the demographic questionnaire included age (years), sex (male or female), educational levels (under diploma, diploma and academic), duration of marriage (years), and duration of treatment (months). Second, participants completed the Adult Trait Hope Scale published in 1991 by Snyder et al. (11). This 12 item self-report instrument contains two subscales: agency and pathway. These components include a sense of personal agency related to goal attainment (4 items) and the ability to recognize/ generate pathways to reach a goal (4 items). This questionnaire also includes 4 items which do not belong to either of the above dimensions. The items are answered by an 8-option Likert scale. Each subscale has a score that ranges from 4 to 32. Hope is scored from 8 to 64, so that higher scores indicate a higher level of agency, pathway, and hope, respectively. We have used the Persian version of hope (Snyder), validated in 2011 by Kermani et al. (12). This version had a Cronbach’s alpha coefficient for reliability of 0.86 and an ICC equal to 0.81. Couples completed the Depression, Anxiety, and Stress Scale (DASS), developed in 1998 by Antony et al. (13). DASS includes 21 items and 3 subscales - anxiety, depression, and stress. The anxiety scale evaluated autonomic arousal, situational anxiety, and subjective experience of anxious affect. The stress scale assessed difficulty relaxing, nervous arousal, and becoming easily upset or agitated, irritable, or over-reactive and impatient (14-16). Each subscale includes 7 items and is categorized according to normal, slight, severe, and very severe. The Persian version of DASS-21 was validated in 2007 by Samani and Joukar (17). The Cronbach’s alpha coefficient to test reliability was 0.81, 0.74, and 0.78, respectively for anxiety, depression and stress. The ICCs equaled 0.80, 0.76, and 0.77, respectively.

We used the SPSS statistical software package (SPSS Inc, Chicago, IL, USA), version 22.0 for statistical analyses. Continues variables were expressed as mean ± SD and categorical variables as number (percentage). Normality of the variables was verified by the Kolmogorov-Smirnov test. The relationship between individual independent variables (demographic, and duration of treatment and marriage) and dependent variables (hope, agency, pathway, anxiety, depression, and stress) were assessed with Pearson correlation coefficient and we conducted the paired t test (between wives and husbands), ANOVA (between groups of infertile in hope subscales), and chi-square test (between groups of infertile in depression, anxiety and stress). Moreover, the mutual effects of demographic characteristics, hope, and groups studied were evaluated using ANCOVA. P<0.05 was considered statistically significant.

## Results

During the study period, 180 infertile couples participated. The mean age was 32.94 ± 4.74 years in men and 29.39 ± 5.09 years in women. Approximately 72 (40%) men and 60 (33.3%) women had academic educations. The mean duration of marriage was 6.07 ± 4.13 years in the couples. The mean duration of treatment was 25.73 ± 25.13 months. The demographic and fertility characteristics of the participants are given in Table 1.

As seen in Table 2, husbands had a significantly higher mean score for hope compared to wives in the normal infertile group (P=0.046). There was no significant difference in the mean score for hope in male and female patients between groups. There was no significant difference in the mean score for agency between wives and husbands in each group. The mean score for agency did not significantly differ in male and female patients between groups. In the normal infertile group, the husbands had a significantly higher mean score for pathway (P=0.032). There was no significant difference observed in the mean score for pathway in male and female patients between groups.

**Table 1:** Demographic and general characteristics of the infertile couples (n=180)


		Oocyte donation[mean ± SD or n (%)]	Embryo donation[mean ± SD or n (%)]	Normal infertile[mean ± SD or n (%)]	P value^*^

Age (Y)	Male	33.05 ± 5.40	32.77 ± 4.51	33.02 ± 4.42	0.912
	Female	30.10 ± 5.41	29.05 ± 5.09	29.02 ± 4.77	0.544
P value^**^			<0.000	<0.000	<0.000	
Education	Male				<0.000
	Female				0.006
Under diploma	Male	8 (13.3)	34 (56.7)	7 (11.7)	
	Female	12 (20)	26 (43.3)	12 (20)	
Diploma	Male	26 (43.3)	13 (21.7)	20 (33.3)	
	Female	22 (36.7)	23 (38.3)	25 (41.7)	
Academic	Male	26 (43.3)	13 (21.7)	33 (55)	
	Female	26 (43.3)	11 (18.3)	23 (38.3)	
P value^**^		0.437	0.180	0.019	
Marital duration (Y)		5.37 (3.94)	6.69 (4.27)	6.15 (4.09)	0.003
Treatment time (Months)		25.52 (20)	28.97 (31.10)	22.72 (22.73)	0.272


*; Test for several independent groups and **; Paired test.

**Table 2:** Hope and its subscales in study couples and groups


		Oocyte donation(mean ± SD)	Embryo donation(mean ± SD)	Normal infertile(mean ± SD)	P value^*^

Hope	Male	52.38 ± 6.52	51.82 ± 6.86	53.93 ± 6.36	0.148
	Female	51.78 ± 7.17	50.72 ± 6.00	51.17 ± 7.17	0.735
	P value^**^	0.614	0.440	0.046	
Agency	Male	26.05 ± 3.71	26 ± 2.86	26.69 ± 3.91	0.335
	Female	25.85 ± 3.52	25.62 ± 3.73	25.43 ± 4.37	0.911
	P value^**^	0.470	0.902	0.126	
Pathway	Male	26.33 ± 3.55	25.82 ± 3.87	27.28 ± 3.44	0.090
	Female	26.17	24.87 ± 3.47	25.73 ± 3.75	0.210
	P value^**^	0.735	0.318	0.032	


*; Test for several independent groups and **; Paired test.

As shown in Table 3, the distribution of depression (normal, slight, medium, severe, and very severe) significantly differed in male subjects among all groups (P=0.01). The frequency of anxiety (normal, slight, medium, severe, and very severe) significantly differed in female subjects (P=0.028). The normal infertile group had a significantly different distribution for anxiety between wives and husbands (P=0.006). The frequency of stress (normal, slight, medium, severe, and very severe) significantly differed in male subjects (P=0.048). In the embryo donation group, stress significantly differed between wives and husbands (P=0.002). In the normal infertile group, stress also significantly differed between wives and husbands (P=0.05).

**Table 3:** Depression, Anxiety, and Stress Scale (DASS) and its subscales in studied couples and groups


		Oocyte donation n (%)	Embryo donationn (%)	Normal infertilen (%)	P value^*^

Depression	Male				0.010
	Female				0.716
Normal	Male	37 (61.7)	27 (45)	38 (63.3)	
	Female	34 (56.7)	26 (43.3)	31 (51.7)	
Slight	Male	2 (3.3)	11 (18.3)	11 (18.3)	
	Female	7 (11.7)	15 (25)	9 (15)	
Medium	Male	11 (18.3)	17 (28.3)	5 (8.3)	
	Female	12 (20)	14 (23.3)	13 (21.7)	
Severe	Male	7 (11.7)	5 (8.3)	3 (5)	
	Female	4 (6.7)	2 (3.3)	4 (6.7)	
Very severe	Male	3 (5)	0 (0)	3 (5)	
	Female	3 (5)	3 (5)	3 (5)	
P value^*^^*^		0.914	0.797	0.091	
Anxiety	Male				0.231
	Female				0.028
Normal	Male	23 (36.3)	25 (41.7)	31 (51.7)	
	Female	24 (40)	26 (43.3)	20 (33.3)	
Slight	Male	3 (5)	4 (6.7)	7 (11.7)	
	Female	7 (11.7)	8 (13.3)	2 (3.3)	
Medium	Male	13 (21.7)	17 (28.3)	13 (21.7)	
	Female	15 (25)	11 (18.3)	17 (28.3)	
Severe	Male	9 (15)	10 (16.7)	5 (8.3)	
	Female	2 (3.3)	3 (5)	12 (20)	
Very severe	Male	0 (0)	0 (0)	0 (0)	
	Female	0 (0)	0 (0)	0 (0)	
P value^*^^*^		1.000	0.938	0.006	
Stress	Male				0.048
	Female				0.831
Normal	Male	31 (51.7)	48 (80)	40 (66.7)	
	Female	30 (50)	29 (48.3)	31 (51.7)	
Slight	Male	8 (13.3)	3 (5)	8 (13.3)	
	Female	9 (15)	13 (21.7)	6 (10)	
Medium	Male	11 (18.3)	8 (13.3)	5 (8.3)	
	Female	10 (16.7)	10 (16.7)	13 (21.7)	
Severe	Male	7 (11.7)	1 (1.7)	5 (8.3)	
	Female	7 (11.7)	4 (6.7)	5 (8.3)	
Very severe	Male	3 (5)	0 (0)	2 (3.3)	
	Female	4 (6.7)	4 (6.7)	5 (8.3)	
P value^*^^*^		0.860	0.002	0.050	


*; Test for several independent groups and **; Paired test.

**Table 4:** Correlation between Depression, Anxiety, and Stress Scale (DASS) subscales and hope


	Depression		Anxiety		Stress	
	r	P value	r	P value	r	P value

Agency	-0.319^*^	<0.000	-0.252^*^	<0.000	-0.272^*^	<0.000
Pathway	-0.187^*^	<0.000	-0.203^*^	<0.000	-0.155^*^	<0.000
Hope	-0.276^*^	<0.000	-0.249^*^	<0.000	-0.228^*^	<0.000


*; P<0.05 and r; Pearson correlation coefficient.

**Tble 5:** ANCOVA results regarding the differences among the studied groups


Variable	Source	Sum of squares	DF	Mean square	F

Hope	Sex	73.167	1	73.167	1.702
	Age	105.917	1	105.917	2.464
	Education	14.444	1	14.444	0.336
	Marital duration	14.969	1	14.969	0.348
	Treatment time	152.952	1	152.952	3.558
	Group	47.850	2	23.925	0.556


*; P<0.05, DF; Degree of freedom, and F; F value.

Additionally, bivariate correlations were conducted among the subscales of the DASS and Adult Trait Hope Scale. Agency negatively and significantly correlated with depression, anxiety, and stress (P<0.001). Pathway and hope showed negative, significant correlations with depression, anxiety, and stress (P<0.001, Table 4). Results of the ANCOVA test showed that regardless of demographic variables, the mean differences in hope did not significantly differ between study groups (Table 5).

## Discussion

To the best of our knowledge, this was the first study that measured two components of the Adult Trait Hope Scale, agency and pathway, in Iranian infertile patients undergoing assisted reproduction treatment according to Snyder’s theory. This was the first study that included both infertile men and women (couples). We classified the study subjects into three groups, oocyte donation, embryo donation, and normal infertile, because the main hypothesis was that a difference existed in hope subscales among these groups and between wives and husbands. Mainly, we hypothesized that those who undergo donation (either embryo or oocyte) could show different hope and other psychological properties compared to other infertile participants. However, many studies have investigated hope in the context of chronic diseases, such as cancer (7, 8). The results of these studies have revealed that hope physiologically and emotionally helped patients tolerate the crisis of the disease (18, 19). Hope is considered an essential element in a chronically ill patients’ life and has a high impact on their adaptation to the disease. Patients who have high levels of hope alleviate psychological tensions better through application of more efficient coping strategies such as reevaluation and problem solving, which affect various stages of the disease process (16, 18, 19). For many infertile patients, the effect of infertility and notably of medical therapy is a considerable emotional stress. It has been shown that infertile women undergo more tension, anxiety, depression, self-reproach, and suicide (9, 20). However, another study revealed that hope was one of the main effective factors for successful IVF (21).

Our study also confirmed the findings that husbands had more hope than wives in the normal infertile group. Further investigation into the two components of hope indicated in the normal infertile group a significantly higher mean score of pathway in husbands. The results of this investigation supported findings extracted from other studies that showed strong inverse relations between hope and psychological symptoms in patients who suffer from chronic diseases. Berendes et al. found an association between higher levels of hope and lower levels of depression among cancer patients (8). These findings agreed with prior research where higher hope was related to less depression in mixed cancer populations (22, 23). Our study also indicated reverse correlations between subscales of DASS and the Adult Trait Hope Scale. Increasing levels of hope resulted in anxiety reduction (24). Some researchers also reported that hope was accompanied by reductions in depression symptoms (8, 25). Studies on the effect of psychological and consultative interventions on the psychological disorders and pregnancy outcomes in infertile couples have shown that psychological therapy effectively reduced anxiety and depression, and increased pregnancy rates (16). A positive psychological treatments, hope therapy, can enhance infertile women’s general health and subsequently improve family health. Therefore, hope therapy is recommended for infertile individuals to be offered with assisted reproductive techniques in order to enhance the quality of life and help these individuals cope with their problems (9, 16).

Our study had several limitations. First, an inherent limitation of this study might be its generalizability. We relied on patients who presented to only one center, a referral clinic for infertility treatment in Iran where patients throughout the country come to this center. Second, the cross-sectional nature of the study only allowed for correlations, but not conclusions on causality.

## Conclusion

Our study was the first study to examine Snyder’s construct of hope in a sample of infertile couples. The results suggest that hope may be important in reducing psychological symptoms and psychological adjustment in those with infertility problems who follow medical recommendations more efficiently through better behavioral patterns, which would accelerate recovery. We suggest that psychological counseling sessions (hope therapy) be offered during reproduction cycles.
